# Platelet-Rich Plasma Modulates Gap Junction Functionality and Connexin 43 and 26 Expression During TGF-β1–Induced Fibroblast to Myofibroblast Transition: Clues for Counteracting Fibrosis

**DOI:** 10.3390/cells9051199

**Published:** 2020-05-12

**Authors:** Roberta Squecco, Flaminia Chellini, Eglantina Idrizaj, Alessia Tani, Rachele Garella, Sofia Pancani, Paola Pavan, Franco Bambi, Sandra Zecchi-Orlandini, Chiara Sassoli

**Affiliations:** 1 Department of Experimental and Clinical Medicine, Section of Physiological Sciences, University of Florence, 50134 Florence, Italy; roberta.squecco@unifi.it (R.S.); eglantina.idrizaj@unifi.it (E.I.); rachele.garella@unifi.it (R.G.); 2 Department of Experimental and Clinical Medicine, Section of Anatomy and Histology, University of Florence, 50134 Florence, Italy; flaminia.chellini@unifi.it (F.C.); alessia.tani@unifi.it (A.T.); sofia.pancani@stud.unifi.it (S.P.); sandra.zecchi@unifi.it (S.Z.-O.); 3 Transfusion Medicine and Cell Therapy Unit, "A. Meyer" University Children’s Hospital, 50134 Florence, Italy; paola.pavan@meyer.it (P.P.); franco.bambi@meyer.it (F.B.)

**Keywords:** α-smooth muscle actin, confocal microscopy, connexin 43, connexin 26, fibrosis, gap junctions, myofibroblasts, Platelet-Rich Plasma, skeletal muscle, transforming growth factor (TGF)-β1

## Abstract

Skeletal muscle repair/regeneration may benefit by Platelet-Rich Plasma (PRP) treatment owing to PRP pro-myogenic and anti-fibrotic effects. However, PRP anti-fibrotic action remains controversial. Here, we extended our previous researches on the inhibitory effects of PRP on in vitro transforming growth factor (TGF)-β1-induced differentiation of fibroblasts into myofibroblasts, the effector cells of fibrosis, focusing on gap junction (GJ) intercellular communication. The myofibroblastic phenotype was evaluated by cell shape analysis, confocal fluorescence microscopy and Western blotting analyses of α-smooth muscle actin and type-1 collagen expression, and electrophysiological recordings of resting membrane potential, resistance, and capacitance. PRP negatively regulated myofibroblast differentiation by modifying all the assessed parameters. Notably, myofibroblast pairs showed an increase of voltage-dependent GJ functionality paralleled by connexin (Cx) 43 expression increase. TGF-β1-treated cells, when exposed to a GJ blocker, or silenced for Cx43 expression, failed to differentiate towards myofibroblasts. Although a minority, myofibroblast pairs also showed not-voltage-dependent GJ currents and coherently Cx26 expression. PRP abolished the TGF-β1-induced voltage-dependent GJ current appearance while preventing Cx43 increase and promoting Cx26 expression. This study adds insights into molecular and functional mechanisms regulating fibroblast-myofibroblast transition and supports the anti-fibrotic potential of PRP, demonstrating the ability of this product to hamper myofibroblast generation targeting GJs.

## 1. Introduction

Adult skeletal muscle can efficiently repair/regenerate after focal damages [1]. Several studies showed that many different cell types endowed with inducible myogenic potential, residing within the muscle tissue or recruited via the blood, might contribute to the formation of nascent contractile myofibers [2,3,4,5,6]. Nevertheless, muscle resident satellite cells are widely recognized as the main players in the repair/regenerative processes [7,8]. After focal injuries, satellite cells undergo activation to essentially recapitulate the steps of embryonic and fetal myogenesis forming new myofibers or fusing with injured myofibers to repair the damage [1,9]. To accomplish their task, satellite cells (but even the myogenic non-satellite cells), require the establishment of a suitable and conductive surrounding microenvironment. This essentially includes pro-myogenic factors, biochemical and physical pro-myogenic signals, juxtacrine, and paracrine interaction with different interstitial nursing cells and a spatially and temporally limited reparative fibrotic response [1,10,11,12,13,14,15]. 

### 1.1. Fibrotic Response in Skeletal Muscle

The activation of fibrogenic pathways represents an adaptive physiological response of tissues, including skeletal muscle, to damage. A crucial process in such a fibrotic response is represented by the differentiation of fibroblasts resident in the extracellular matrix (ECM) towards myofibroblasts [16]. This is essentially promoted by the combined action of pro-fibrogenic agents, such as transforming growth factor (TGF)-β1, mainly released by infiltrating inflammatory cells (particularly macrophages) at the site of the injury and by the fibroblasts/myofibroblasts itself, and mechanical stimuli coming from the damaged microenvironment [16,17]. Myofibroblasts are characterized by a prominent rough endoplasmic reticulum, typical of collagen-synthetically active fibroblasts, by the de novo expression of α-smooth muscle actin (α-sma) within well-assembled stress fibers that confers contractile properties to the cells. Stress fibers are anchored to fibronexus, a specialized focal adhesion complex on the myofibroblast surface, to link intracellular actin filaments with extracellular fibronectin fibrils. Through fibronexus, the force generated by stress fibers can be transmitted to the surrounding ECM, and vice-versa the ECM mechanical signals can be transduced via this mechano-transduction system into intracellular signals [18]. Moreover, even if myofibroblasts are not regarded as electrically excitable cells, they show peculiar biophysical properties and trans-membrane ion currents typical of smooth muscle cells. In this regard, it has been reported that human atrial myofibroblasts can express a Na^+^ current (I_Na_) and biophysical properties that could give rise to regenerative action potentials [19,20]. In addition, myofibroblasts typically show the inward-rectifier K^+^ current (I_kir_), which especially increases under TGF-β1-treatment [21,22,23,24]. In physiological conditions, the permanence and function of myofibroblasts after muscle damage are temporally and spatially limited. Indeed, they are responsible for the deposition of ECM components to form a transient contractile scar essentially required to rapidly restore tissue integrity and preserve muscle function, to support activated SCs and the nascent myofibers mechanically. Once the tissue regeneration has taken place, the scar will be degraded thanks to the balanced and finely tuned activity of proteolytic enzymes selectively digesting individual components of ECM, namely matrix metalloproteinases (MMPs), and of their specific tissue inhibitors (TIMPs), that are mainly secreted by different cells including, among others, fibroblasts and inflammatory cells [25]. Myofibroblasts progressively disappear, undergoing apoptosis and/or senescence or reverting to a quiescent state [26,27]. By contrast, the persistence of myofibroblasts in an activated state has been associated with an aberrant maladaptive reparative response to chronic or extended damage leading to the formation of a permanent scar replacing the normal functional tissue and hampering the endogenous cell mediated-mechanism of muscle regeneration [10,16,17,28,29]. Therefore, therapies aimed to limit myofibroblast generation and functionality may result strategical and effective for preventing tissue fibrosis development and thus promoting the regeneration of damaged muscles. 

### 1.2. PRP as an Anti-fibrotic Agent

In this regard, Platelet-Rich Plasma (PRP)—defined as a plasma fraction with a concentration of platelets above baseline levels and representing a source of numerous biologically active molecules—may offer promising perspectives [30]. Indeed, many in vitro and in vivo studies have demonstrated the anti-fibrotic potential of this blood product in different tissues [31,32,33,34,35,36,37,38], including skeletal muscle [30,39,40,41,42,43,44,45,46], and have indicated the fibroblast-myofibroblast transition as the cell process target of its action [31,32,33,36,38,44,47,48]. Furthermore, the positive contribution of PRP to skeletal muscle regeneration has been demonstrated either in vivo or in vitro, thanks to its capability to promote the myogenic program [30]. Nevertheless, the anti-fibrotic potential of PRP needs to be investigated more in-depth, and the molecular targets of the action of this plasma product need to be clearly identified. 

### 1.3. Gap Junction Intercellular Communication (GJIC)

The gap junction (GJ) channels are dynamic membrane domains built of two docking hemichannels called connexons assembled in the plasma membranes of two adjacent cells. Each connexon is a hexameric structure consisting of six transmembrane proteins named connexins (Cxs) that may have different molecular weights [49] and form an aqueous pore. The opening of these channels allows the flow of ions and small molecules (less than 1000 MW molecular weight size fractions) such as sugars, amino acids, oxygen, as well as second messengers such as cAMP, inositol phosphates, and calcium directly from one cell to another. The type of molecules (second messengers) passing through GJs can be influenced by the Cx isoform composition of the GJ channels. When the Cx isoforms are of the same type within a hemichannel, the resulting structure is called homomeric, whereas it is called heteromeric if more than one Cx isoform is present. The GJ channels composed of two identical hemichannels are named homotypic, and those consisting of two different hemichannels are named heterotypic. These two types of GJ channels exhibit peculiar and different gating properties influencing their voltage sensitivity [50]. GJIC is proposed to play a role in regulating the fibroblasts transition towards myofibroblasts as well as to be involved in the functional coupling of myofibroblasts to coordinate their activity [18,51,52,53,54]; however, GJ channel functionality and Cx composition during the phenotypic progression of fibroblasts into the myofibroblasts have not been fully elucidated yet and deserve more attention. 

In the present in vitro study, by combining morphological, biomolecular, biochemical, and electrophysiological analyses, we extended our previous researches further exploring the potential molecular targets of the inhibitory action of PRP on myofibroblast generation. In particular, we focused the attention on the GJIC. The experimental model to evaluate fibroblast to myofibroblast transition has been previously validated [23,24,48,55,56,57] and consists in the culture of the cells in low serum conditions in the presence of TGF-β1. The treatment with PRP was also conducted as previously reported [48,57,58]. 

Here, while confirming the anti-fibrotic potential of PRP we provide the first experimental evidence that voltage-dependent GJ functionality and the expression of Cx43, a typical Cx forming voltage-dependent connexons, are important mechanisms by which TGF-β1 endorses fibroblasts differentiation towards myofibroblasts, and that PRP treatment hampers this effect. Moreover, we also demonstrated the involvement of not-voltage dependent GJs and Cx26, a typical Cx type forming not (or at least, scarcely) voltage-dependent connexons.

## 2. Materials and Methods

### 2.1. Platelet-Rich Plasma (PRP) Preparation

For the present experiments, thawed ready-to-use activated PRP aliquots classified as not suitable for transfusion-infusion purposes previously prepared and stored at −80 °C were used [48]. Briefly, PRP was collected from the blood of healthy adult donors subjected to plasma-platelet apheresis (Haemonetics MCS®, Haemonetics, Milan, Italy) as previously reported in detail [57]. The final platelet concentration in each PRP (without leukocytes) aliquot was 2 × 10^6^ platelets/µL. Platelet activation was induced by the addition of a calcium digluconate solution (10%). The donors gave their written informed consent to allow the use of PRP for in vitro experimentations for which the Ethical Committee’s approval is not required. PRP treatment was performed as previously reported [48,57].

### 2.2. Cell Culture and Treatments 

Murine NIH/3T3 fibroblastic cells were obtained from American Type Culture Collection (ATCC, Manassas, VA, USA). The cells were grown in proliferation medium (PM), consisting of Dulbecco’s Modified Eagle’s Medium (DMEM; Sigma, Milan, Italy) containing 4.5 g/L glucose supplemented with 10% fetal bovine serum (FBS) and 1% penicillin/streptomycin (Sigma), at 37 °C in a humidified atmosphere of 5% CO_2_. Fibroblastic cells were induced to differentiate into myofibroblasts by shifting them in differentiation medium (DM) consisting of DMEM supplemented with 2% FBS and 2 ng/mL TGF-β1 (PeproTech, Inc., Rocky Hill, NJ, USA) for 48 h and 72 h, as previously reported [48]. In parallel experiments, to estimate the influence of PRP on fibroblast-myofibroblast transition, PRP was added to DM (1:50) [48,57]. In some experiments, the cells were cultured in PM, DM, or DM + PRP in the presence of 1 mM heptanol (Sigma), a specific GJ blocker, to evaluate the involvement of GJs in myofibroblastic differentiation.

### 2.3. Electrophysiological Records

Cell pairs were analyzed by the dual whole-cell patch-clamp technique, as previously reported [59,60,61]. Both passive membrane properties and GJ functionality were investigated. To this aim, cells were plated on glass coverslips (50,000 cells on each glass coverslips) to be located in the recording chamber and continuously superfused at a rate of 1.8 ml/min by a Pump 33 (Harvard Apparatus) with a physiological bath solution containing (mM) 140 NaCl, 5.4 KCl, 1.8 CaCl_2_, 1.2 MgCl_2_, 10 D-glucose, and 5 HEPES (pH set at 7.4 with NaOH). The patch electrodes, pulled from borosilicate glass (GC 150–15; Clark, Reading, UK), were filled with the following solution (mM): 130  KCl, 10  NaH_2_PO_4_, 0.2  CaCl_2_, 1  EGTA, 5  MgATP, and 10  HEPES (pH was set to 7.2 with KOH). When filled, the pipette resistance ranged between 1.5 to 3.0 MΩ. Experiments were achieved at room temperature (22 °C). The set up for electrophysiological measurements was as previously reported [61] and consisted of the Axopatch 200 B amplifier (Axon Instruments, Union City, CA), an analog-to-digital/digital-to-analog interface (Digidata 1200; Axon Instruments), and pClamp 6 software (Axon Instruments). Currents were low-pass filtered at 1 kHz with a Bessel filter. The passive membrane properties, membrane resistance (R_m_), and membrane linear capacitance (C_m_) were consistently estimated in voltage-clamp starting from a holding potential (HP) of -70 mV and applying a 10-mV positive and negative step pulse. In brief, R_m_ was calculated using the relation: R_m_ = (ΔV − I_m_R_a_)/I_m_, where ΔV is the command voltage step amplitude, I_m_ is the steady-state membrane current, and R_a_ the access resistance [23,62]. C_m_ was calculated from C_m_ = ΔQ(R_m_ + R_a_)/R_m_ΔV, corrected according to a previous report [63]. To properly compare the currents recorded from different cells, their values were normalized to C_m_, assuming that the specific C_m_ was constant at 1 μF/cm^2^. The ratio I/C_m_ was intended as current density (in pA/pF). The junction potential of the electrode was estimated before making the patch (about −10 mV) and then was subtracted from the recorded membrane potential. The resting membrane potential (RMP) was recorded in current-clamp mode with a stimulus waveform: I = 0 pA. The protocol of stimulation used to record the currents flowing through GJs in voltage clamp and the recording procedure have been previously reported [61,64,65]. In brief, cell 1 of the pair was stepped from a holding potential (HP) of 0 mV, using a bipolar 5 s pulse protocol starting at trans-junctional voltage Vj = ±10 mV and ongoing at 20 mV increments up to ± 150 mV. The transjunctional current flowing through GJs is indicated as I_j_. Precisely, the amplitude of I_j_ determined at the peak was named I_j,inst_ (instantaneous transjunctional current), whereas that measured at the end of each pulse is indicated as I_j,ss_ (steady-state transjunctional current). These values were used to calculate the related gap junctional conductances, G_j,inst_ and G_j,ss_, by the ratios: G_j,inst_ = I_j,inst_/V_j_ and G_j,ss_ = I_j,ss_/V_j_, respectively. The mean values of G_j,ss_ were normalized to those of G_j,inst_, plotted as a function of V_j_ and fitted, when possible, with a Boltzmann function using the equation: G_j_ = (G_max_ − G_min_)/(1 + exp (−A(V_j_ − V_0_))) + G_min_. In a set of experiments, heptanol (1 mM) was acutely applied to the bath solution to block gap junctional currents. 

### 2.4. Silencing of Cx43 Expression by Short Interfering RNA 

To inhibit the expression of Cx43, the cells were cultured either in a 6-wells/plate or on sterile glass coverslips put on the bottom of a 6-wells/plate in PM till a confluence of 80% and then transfected with a mix of short interfering RNA duplexes (siRNA; Santa Cruz Biotechnology, Santa Cruz, CA) corresponding to 3 distinct regions of the DNA sequence of mouse Cx43 gene (NM_010288): 5′CCCAACUGAACCUUAAGAA3′, 5′CCUCACCAAAUGAUUUCUA3′, and 5′CCUACCAGUUUCUUCAAGU3′ and/or with a non-specific scrambled (SCR)-siRNA (Santa Cruz Biotechnology) used as control. The siRNA transfections were performed according to manufacturer’s instructions (Santa Cruz Biotechnology) and as previously reported [59]. Briefly, the cells were transfected with Cx43-siRNA duplexes or SCR-siRNA (20 nM) for 24 h and then shifted in fresh PM for additional 5 h. Thereafter, the transfected cells were cultured in DM with the addition or not of PRP for 48 h before being processed for Western blotting or immunofluorescence analysis of Cx43 and α-sma.

### 2.5. Reverse Transcription - Polymerase Chain Reaction (RT-PCR)

Cellular expression levels of Cx43 were evaluated by RT-PCR, as previously reported [48]. Briefly, according to manufacturer’s instructions, total RNA was extracted from the cells cultured in the different experimental conditions on the wells of 6-wells/plates, by using TRIzol Reagent (Invitrogen, Life Technologies, Grand Island, NY, USA). One µg of total extracted RNA was reverse transcribed and amplified by using SuperScript One-Step RT-PCR System (Invitrogen, Life Technologies). cDNA synthesis was performed at 55 °C for 30 min; the samples were pre-denatured at 94 °C for 2 min and then subjected to 40 cycles of PCR performed at 94 °C for 15 s, alternating with 55 °C for 30 s and 72 °C for 1 min; the final extension step was performed at 72 °C for 5 min. The mouse gene-specific primers used were as follow: Cx43 (X61576.1), forward 5′-AACAGTCTGCCTTTCGCTGT-3′ and reverse 5′-ATCTTCACCTTGCCGTGTTC-3’; β-actin (NM_007393), forward 5′-ACTGGGACGACATGGAGAAG-3′ and reverse 5′-ACCAGAGGCATACAGGGACA-3′. β-actin mRNA was used as an internal standard. Blank controls, consisting of no template (water), were performed in each run. The amplified samples were electrophoresed on 1.8% agarose gel containing ethidium bromide staining, and the intensity of the related bands was quantified by densitometric analysis by using ImageJ 1.49v software (NIH, https://imagej.nih.gov/ij/). Each band intensity was normalized to the relative β-actin.

### 2.6. Confocal Laser Scanning Microscopy 

Cells grown on sterile glass coverslips in the different experimental conditions were fixed with paraformaldehyde (PFA) 0.5% diluted in PBS for 10 min at room temperature. Fixed cells were washed and permeabilized with cold acetone for 3 min, incubated with a blocking solution containing 0.5% bovine serum albumin (BSA, Sigma) and 3% glycerol in PBS for 20 min and thereafter incubated overnight at 4 °C with the following antibodies: mouse monoclonal anti-α-sma (1:100; Abcam, Cambridge, UK), rabbit polyclonal anti-Cx43 (1:250; Chemicon, Temecula, CA, USA), rabbit polyclonal anti-type-1 collagen (1:50; Santa Cruz Biotechnology), or mouse monoclonal anti-Cx26 (1:50; Sigma). The immunoreactions were revealed by specific anti-mouse Alexa Fluor 488- or 568 conjugated IgG or anti-rabbit Alexa Fluor 488- conjugated IgG (1:200; Molecular Probes, Eugene, OR, USA). In some experiments the fixed cells were incubated with Alexa Fluor 488-conjugated wheat germ agglutinin (WGA, 1:100; Molecular Probes) for 10 min at room temperature, which binds glycoconjugates present on cell membranes, or counterstained with propidium iodide (PI, 1:30 for 30 s; Molecular Probes), to detect nuclei. Negative controls were carried out by replacing the primary antibodies with non-immune serum, while cross-reactivity of the secondary antibodies was evaluated in control experiments in which primary antibodies were omitted. The immunolabeled samples were washed and mounted with an antifade mounting medium (Biomeda Gel mount, Electron Microscopy Sciences, Foster City, CA, USA) to allow the observation under a confocal Leica TCS SP5 microscope equipped with a HeNe/Ar laser source for fluorescence measurements and differential interference contrast (DIC) optics (Leica Microsystems, Mannheim, Germany). Observations were performed by means of a Leica Plan Apo 63×/1.43NA oil immersion objective. A series of optical sections (1024 × 1024 pixels each; pixel size 204.3 nm) 0.4 μm in thickness were taken throughout the depth of the cells preparations at intervals of 0.4 μm, and the images were projected onto a single ‘extended focus’ image. Densitometric analyses of the intensity of α-sma, type-1 collagen, Cx43 and Cx26 fluorescence signals were performed on digitized images using ImageJ 1.49v software (NIH, https://imagej.nih.gov/ij/) in 20 regions of interest (ROI) of 100 μm^2^ for each confocal stack (at least 10). 

### 2.7. Western Blotting 

Total proteins extracted from the cells in the different experimental conditions were quantified, as reported previously [48]. Forty µg of total proteins were subjected to electrophoresis on NuPAGE® 4%–12% Bis-Tris Gel (Invitrogen, Life Technologies; 200 V, 40 min) and blotted onto polyvinylidene difluoride (PVDF) membranes (Invitrogen, Life Technologies; 30 V, 1 h). The membranes were incubated with mouse monoclonal anti-α-sma (1:1000; Abcam), rabbit polyclonal anti-Cx43 (1:2500; Chemicon), and mouse monoclonal anti-Cx26 (1:500; Sigma) overnight at 4 °C. Immunodetection was performed according to the Western Breeze® Chromogenic Western Blot Immunodetection Kit protocol (Invitrogen, Life Technologies). The same membranes were subjected to the immunodetection of the expression of α-tubulin (rabbit polyclonal anti α-tubulin, 1:1000; Merck, Milan, Italy), assumed as control invariant protein. Densitometric analysis of the bands was performed using ImageJ 1.49v software (NIH, https://imagej.nih.gov/ij/), and the values normalized to control. 

### 2.8. Statistical Analysis

Data were expressed as means ± standard error of the mean (S.E.M.) as a result of at least 3 independent experiments performed in triplicate. A 95% confidence level was used, assuming a normal distribution of values. Unpaired Student’s t-test was used to compare the means of two conditions for independent data, statistically. The one-way analysis of variance (ANOVA) for any single independent variable was used to compare the differences between more than 2 groups and was followed by Tukey HSD or Bonferroni’s post hoc adjustment. In electrophysiological experiments, ‘n’ indicates the number of cells analyzed. Values of *p* < 0.05 were considered statistically significant. Calculations were performed using GraphPad Prism software program (GraphPad, San Diego, CA, USA) and Microsoft Office Excel 2013 (Microsoft Corporation, Redmond, WA, USA).

## 3. Results

### 3.1. PRP Prevented TGF-β1- Induced Fibroblast to Myofibroblast Transition 

**Figure 1:**
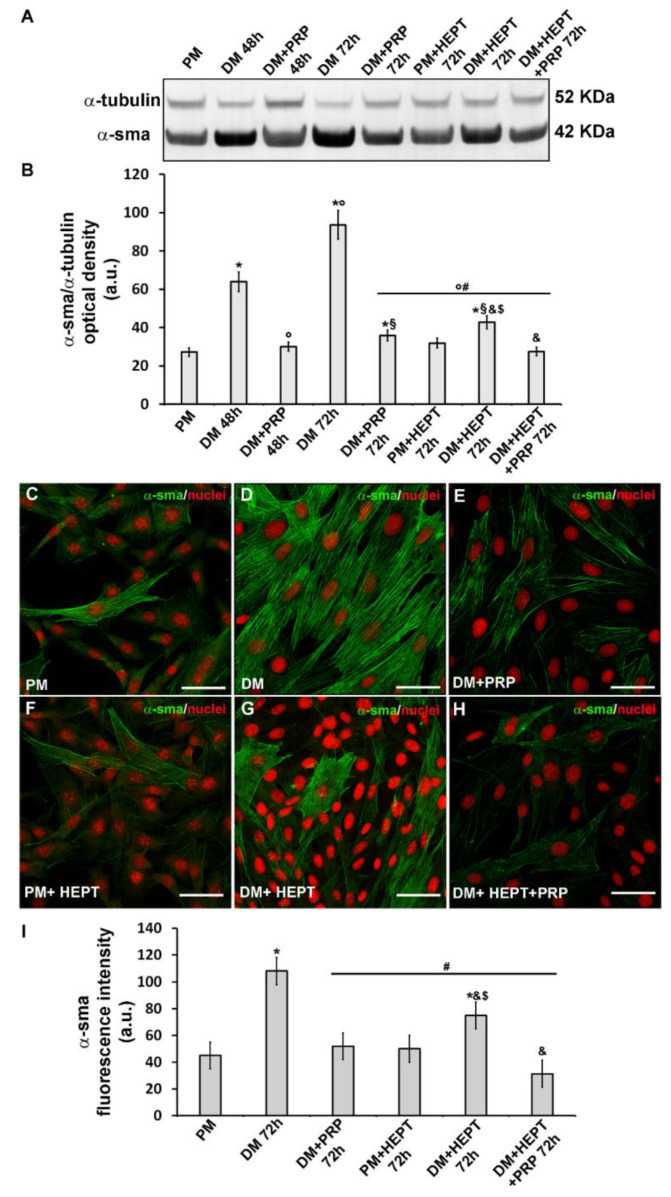
Evaluation of the effects PRP on fibroblast to myofibroblast transition and of the involvement of GJs: α-sma expression. Fibroblasts were induced to differentiate into myofibroblasts by culturing in differentiation medium (DM) in the presence or absence of PRP for 48 h and 72 h. Cells cultured in proliferation medium (PM) served as control undifferentiated cells. In parallel experiments, fibroblasts were cultured in PM or in DM in the presence of heptanol (HEPT), a common GJ channel blocker, in the presence or absence of PRP for 72 h. (**A**,**B**) Western Blotting analysis of α-sma expression. (**A**) Representative Blot. (**B**) Histogram showing the densitometric analysis of the bands normalized to α-tubulin. (**C**–**H**) Representative confocal fluorescence images of the cells immunostained with antibodies against α-sma (green) and counterstained with propidium iodide (PI) to detect nuclei. Scale bar: 50 µm. (**I**) Histogram showing the densitometric analysis of the intensity of the α-sma fluorescence signal performed on digitized images in 20 regions of interest (ROI) of 100 μm^2^ for each confocal stack (10). Data shown are mean ± S.E.M. and represent the results of at least three independent experiments performed in triplicate. Significance of difference: * *p* < 0.05 versus PM; ° *p* < 0.05 versus DM 48 h; # *p* < 0.05 versus DM 72 h; § *p* < 0.05 versus DM + PRP 48 h; & *p* < 0.05 versus DM + PRP 72 h; $ *p* < 0.05 versus PM + HEPT 72 h (One-way ANOVA followed by the Tukey post hoc test).

Successful in vitro differentiation of NIH/3T3 fibroblasts towards myofibroblasts induced by the well-known pro-fibrotic factor TGF-β1 and the ability of PRP to prevent this transition were confirmed by morphological, biochemical and electrophysiological evaluations. Fibroblasts induced to differentiate by culturing in DM exhibited the typical features of myofibroblastic phenotype. Indeed, as judged by Western blotting analysis, they showed a significant increase of the expression of α-sma (*p* < 0.05), the most reliable marker of myofibroblasts, after 48 h and even more after 72 h of culture, as compared to control undifferentiated cells in PM (Figure 1A,B). Moreover, the immunocytochemical analysis at confocal microscopy, performed after 72 h of culture, confirmed the data of Western blotting and showed that this protein was well organized along filamentous structures (Figure 1C,D,I). 

**Figure 2:**
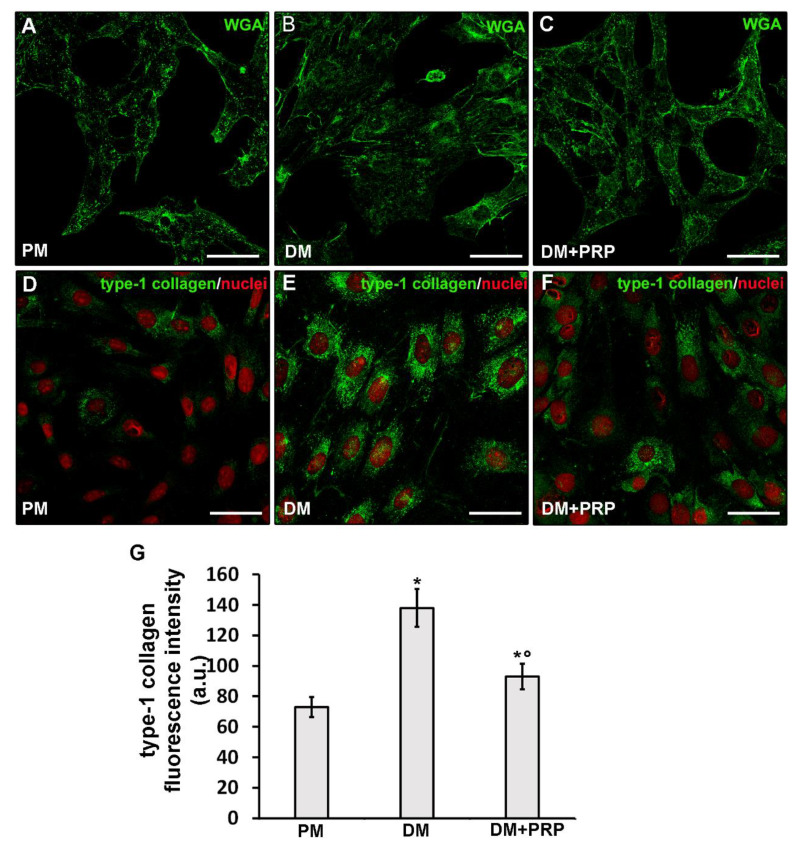
Effects of PRP on fibroblast to myofibroblast transition: Cell morphology and type-1 collagen expression. Fibroblasts were induced to differentiate into myofibroblasts by culturing in differentiation medium (DM) in the presence or absence of PRP for 72 h. The cells cultured in proliferation medium (PM) served as control undifferentiated cells. (**A**–**F**) Representative confocal fluorescence images of the cells (**A**–**C**) stained with Alexa Fluor 488-conjugated WGA (green) to reveal the plasma membrane and (**D**–**F**) immunostained with antibodies against type-1 collagen (green) and counterstained with propidium iodide (PI), to label nuclei. Scale bar: 50 µm. (**G**) Histogram showing the densitometric analysis of the intensity of type-1 collagen fluorescence signal performed on digitized images in 20 regions of interest (ROI) of 100 μm^2^ for each confocal stack (10). Data are reported as mean ± S.E.M. and represent the results of at least three independent experiments performed in triplicate. Significance of difference: * *p* < 0.05 versus PM; ° *p* <0.05 versus DM (One-way ANOVA followed by the Tukey post hoc test).

Moreover, cells cultured in DM for 72 h, appeared much larger with a more polygonal shape as compared to the cells cultured in PM which, instead, were smaller and spindle-shaped as judged by the confocal fluorescence analysis after labeling with the membrane dye Alexa Fluor 488 conjugated WGA (Figure 2A,B). Differentiated cells also showed a robust increase (*p* < 0.05) in the expression of type-1 collagen at the cytoplasmic level and, in some cases, even outside the cells in a filamentous form (Figure 2D,E,G).

**Figure 3:**
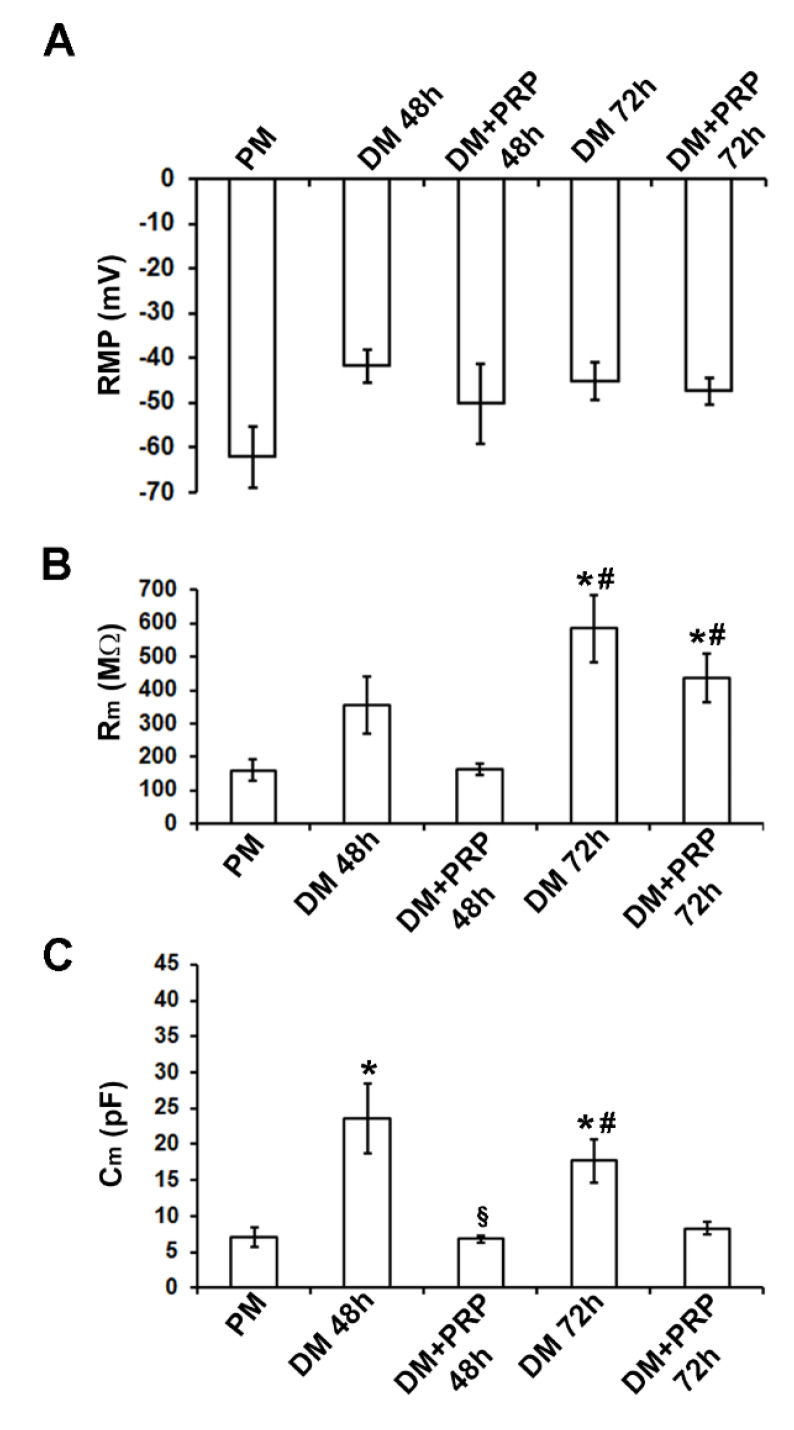
Effects of PRP on fibroblast to myofibroblast transition: Electrophysiological analysis of biophysical properties. (**A**) Resting membrane potential (RMP, in mV) recorded in the different conditions. Myofibroblasts have a tendency to be more depolarized. (**B**) Membrane resistance (R_m_, in MΩ) shows higher values in myofibroblasts grown in differentiation medium (DM) compared to fibroblasts grown in proliferation medium (PM). (**C**) Membrane capacitance (C_m_, in pF): Myofibroblasts show higher values compared to the undifferentiated cells in PM. All values are reported as mean ± S.E.M. and are listed in Table 1. * *p*  <  0.05 versus PM; # *p*  <  0.05 versus DM + PRP 48 h; § *p* < 0.05 versus DM 48 h (one-way ANOVA, followed by Bonferroni’s post hoc test).

The electrophysiological analysis of the passive membrane properties achieved by the whole-cell patch-clamp technique confirmed that the cells cultured in DM acquired the myofibroblastic phenotype. First, the resting membrane potential, RMP, was recorded and it was found that the values recorded from the cells cultured in DM for 48 h tended to be more depolarized compared to control undifferentiated fibroblasts in PM, in accordance with previous observations [23,24]. The overall results from all of the experiments done are shown in Figure 3A and Table 1. The statistical analysis of the RMP values between the different conditions was achieved with one–way ANOVA that provided overall results for our data. Despite the observed tendency to depolarization, the differences between the means did not turned out to be statistically significant (*p* = 0.26; F = 1.45 < Fcrit = 3.15; df = 30). 

We then analyzed the cell membrane resistance, R_m_, in the voltage-clamp mode of our device. As shown in Figure 3B, R_m_ increased after 48 h and even more after 72 h of culture in DM. The one-way ANOVA analysis of R_m_ indicated statistical significance (*p* = 0.00010; F = 8.99 > Fcrit = 2.83; df = 50). To know which groups were significantly different from another, we used the Bonferroni post-hoc test. The resulting significance is indicated by the symbols depicted in Figure 3B and Table 1. 

Similarly, the cell capacitance, C_m_, of cells cultured in DM, usually assumed as an index of cell surface, changed significantly (*p* = 0.0085; F = 4.52 > Fcrit = 2.86; df = 45; one way ANOVA). It tended to increase compared to that estimated in PM (Figure 3C; Table 1), being (*p* < 0.05) higher for cells in DM, especially after 48 h. These results were consistent with the observed cell morphology (Figure 2A,B).

The treatment with PRP actually counteracted the TGF-β1-induced fibroblast to myofibroblast transition. Indeed, the cells cultured in DM + PRP exhibited a significant (*p* < 0.05) reduction of α-sma (Figure 1A,B,E,I) with respect to differentiated cells in DM, together with different morphology, more similar to that of cells cultured in PM (Figure 2C) and a significantly reduced expression of type-1 collagen (*p* < 0.05) (Figure 2F,G).

Of interest, the electrophysiological analyses performed on the cells cultured in DM + PRP for the first time, revealed that R_m_ values tended to decrease compared to those measured in DM (*p* > 0.05) both after 48 h and 72 h of culture (Figure 3B; Table 1). As well, C_m_ values evaluated from cells cultured in DM + PRP were significantly reduced (*p* < 0.05) compared to those in DM, consistent with the observed changed morphology of these cells (Figure 3C; Table 1).

### 3.2. PRP Modifies Transjunctional Currents (I_j_) and Gap Junctional Conductance (G_j_) in Myofibroblast Pairs 

**Figure 4:**
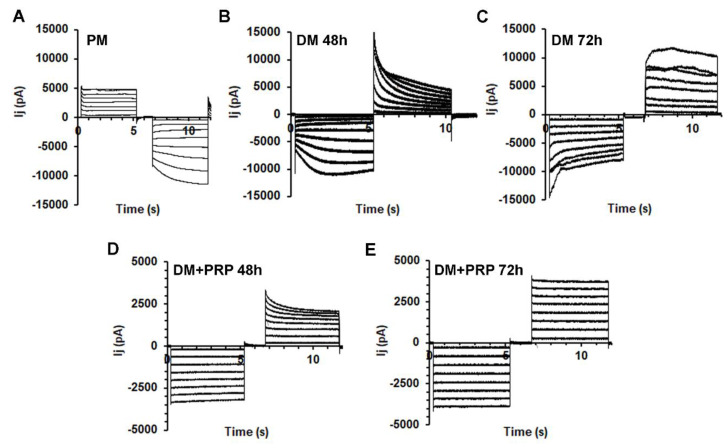
Time course of the transjunctional currents I_j_, recorded from fibroblast and myofibroblast pairs in the absence or presence of PRP. (**A**) Representative I_j_ tracings (in pA) recorded in response to a bipolar pulse protocol applied to a fibroblast pair cultured in proliferation medium (PM). Note the asymmetrical time course between the two voltage polarities with a linear response for positive V_j_. (**B**,**C**) Typical asymmetrical and almost voltage-dependent I_j_ tracings recorded from (**B**) a myofibroblast pair cultured in differentiation medium (DM) for 48 h and from (**C**) a myofibroblast pair in DM for 72 h. (**D**,**E**) Representative I_j_ recorded from a cell pair grown in (**D**) DM with PRP for 48 h (DM + PRP 48 h) and (**E**) for 72 h (DM + PRP 72 h). Note the completely linear and symmetrical responses in the latter condition.

Next, the transjunctional currents (I_j_) were analyzed in cell pairs in different experimental conditions by the dual whole-cell technique. Most of the fibroblast pairs cultured in PM exhibited families of I_j_ current traces with a nearly heterogeneous time course. Typical tracings obtained from a not differentiated cell pair cultured in PM are depicted in Figure 4A.

A minority of the cell pairs cultured in PM (about 20%) showed current records with a symmetrical time course for negative and positive V_j_ (not shown), indicating the involvement of homotypic GJs. Moreover, they also showed a linear I_j_-V_j_ plot, suggesting the presence of not-voltage-dependent connexons. By contrast, the remaining 80% of the cell pairs investigated showed a non-linear time course. In particular, only 40% of these cells showed a symmetrical voltage dependence for positive and negative V_j_, suggesting the involvement of homotypic GJs and voltage-dependent connexons; 60% of the cells showed asymmetrical voltage dependence (Figure 4A). This may suggest the dominant presence of heterotypic GJs in control not differentiating fibroblasts.

We then analyzed the time course of the I_j_ evoked in myofibroblast pairs. Typical tracings of the I_j_ evoked in cell pairs cultured in DM for 48 h or 72 h are shown in Figure 4B,C, respectively. About 25% of these cells showed linear not-voltage-dependent I_j_. Notably, about 75% of the cell pairs cultured in DM for 48 h exhibited a non-linear time course, suggesting the prevalent expression of voltage-dependent connexons. Only 33% of this kind of cell pairs showed an asymmetrical voltage-dependence suggesting a minor presence of heterotypic voltage-dependent GJs in differentiating myofibroblasts. In contrast, 67% of this kind of response was symmetrical for negative and positive V_j_, suggesting that the majority of the GJs involved in this myofibroblastic population were voltage-dependent and homotypic. 

After 72 h of culture in DM, we could observe an increase of the percentage of cell pairs with symmetrical voltage-dependent responses (about 80% in 72 h versus 67% in 48 h), indicating a progressive increase in the number of myofibroblasts exhibiting voltage-dependent and homotypic GJs.

**Figure 5:**
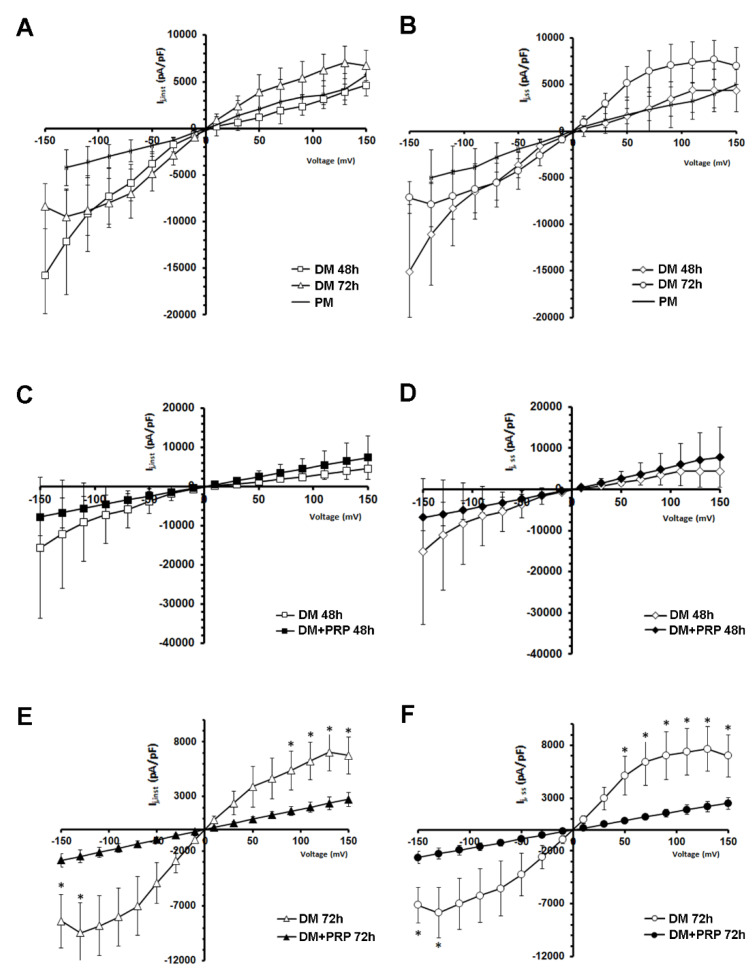
Voltage dependence of the transjunctional currents I_j_, recorded from fibroblast and myofibroblast pairs in the absence or presence of PRP. (**A**–**F**) Transjunctional current values normalized for cell capacitance (in pA/pF), recorded from all of the fibroblast pairs cultured in proliferation medium (PM, continuous line, *n* = 6), and in differentiation medium (DM) for 48 h (*n* = 9) and 72 h (*n* = 8) plotted versus V_j_. Panels A, C, and E show the I_j,inst_ values whereas panels B, D, and F show the I_j,ss_ values. Note that the plots related to DM show different slopes. (**C**,**D**) Comparison between I_j_-V_j_ plot obtained from cell pairs cultured in DM for 48 h (open symbols, *n* = 9) and from cell pairs cultured in DM + PRP for 48 h (filled symbols, *n* = 9). Adding PRP to the culture medium for 48 h, altered the I_j_ voltage dependence, causing an almost complete linearity with voltage both for I_j,inst_ and I_j,ss_. (**E**,**F**) I_j_-V_j_ plots related to 72 h treatments. The presence of PRP in DM for 72 h (DM + PRP 72 h, filled symbols, *n* = 14), strongly reduced the mean normalized current amplitude observed in DM 72 h (open symbols, *n* = 8) and altered the I_j_ voltage dependence, causing an almost complete linearity with voltage both for I_j,inst_ and I_j,ss_. All values represent mean  ±  S.E.M. * *p*  <  0.05 (unpaired Student’s t-test).

The voltage dependence of I_j_ was evaluated by plotting the mean values of instantaneous currents, I_j,inst_, (Figure 5A,C,E) and the steady-state currents, I_j,ss_, (Figure 5B,D,F) as a function of V_j_ (I_j_-V_j_ plot). 

Notably, from a qualitative point of view, the I_j_-V_j_ plots showed a different shape according to the different culture conditions, namely PM and DM 48 h and 72 h (Figure 5A,B). Ij currents recorded in PM showed the smallest amplitude and a scarce deviation from linearity, especially for negative V_j_, showing a similar slope for both the V_j_ polarities (Figure 5A,B). In contrast, the plot related to DM 48 h was almost linear and smoother for positive V_j_ (Figure 5A,B). The plot related to DM 72 h showed a kind of shoulder becoming S-shaped (Figure 5A,B). For negative V_j_ the resulting I_j_-V_j_ plots showed a different steepness compared to that observed for positive V_j_, and it was similar at any time in culture in DM for 48 h and 72 h (Figure 5A,B). Again, I_j_ data showing this asymmetrical V_j_ -dependence are indicative of heterotypic GJ channels. The I_j_ evaluated both at the peak (I_j,inst_) and at the steady-state (I_j,ss_) showed a progressively more marked voltage-dependence as the time in culture in DM increased. Based on this observation, we suggest a major involvement of voltage-dependent connexons during the differentiation time. Of note, when cells were cultured in DM + PRP for 48 h, about 75% of the cell pairs showed a linear time course (Figure 4D), even if this kind of response was not perfectly symmetrical for negative and positive V_j_ for all of the cell pairs investigated. When the mean values of all the I_j,inst_ and I_j,ss_ recorded were plotted versus V_j_, the relation resulted approximately linear and symmetrical over the entire voltage range, clearly indicating the prevalence of not voltage-dependent homotypic GJ channels (Figure 5C,D) in this culture condition. On the other hand, the cells cultured in DM + PRP for 72 h exhibited only not-voltage dependent Ij (100%) (Figure 4E). Indeed, they showed a linear response and a perfectly symmetrical time course for positive and negative V_j_. Again, the I_j_-V_j_ plot analysis showed the I_j_ linearity with V_j_ and a marked symmetry, strongly indicating the presence of not-voltage-dependent homotypic GJ channels (Figure 5E,F). Remarkably, for the largest voltage steps applied, the mean current amplitudes recorded from cells cultured in DM for 72 h were statistically different (*p* < 0.05; multiple unpaired Student’s t-test) to those estimated in DM + PRP at the same time. Of note, the comparison of I_j_-V_j_ plots in Figure 5E,F with those in Figure 5C,D indicated that the maximal recorded normalized mean amplitude both of I_j,inst_ and I_j,ss_ resulted smaller in DM + PRP 72 h than in DM + PRP 48 h. For instance, the mean I_j,ss_ value estimated for the +150 mV step pulse was 2508 ± 566 pA/pF in DM + PRP 72 h and 7801 ± 7006 pA/pF in DM + PRP 48 h (Figure 5C,D).

Therefore, the features of I_j_ observed in the cells cultured in DM + PRP, such as the symmetry of the time course and the linearity of I_j,inst_ and I_j,ss_ versus voltage plots lead us to suggest a lessened contribution of voltage-dependent connexons in this condition.

**Figure 6:**
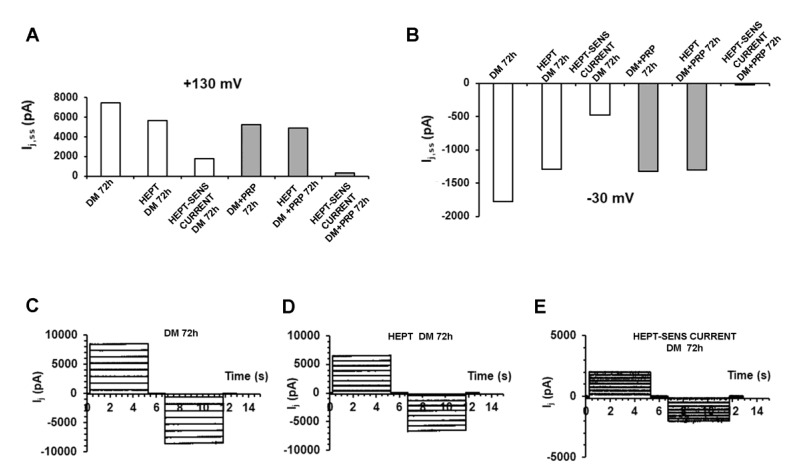
Evaluation of the current flowing through the GJs by acute addition of heptanol under different experimental conditions. (**A**,**B**) Evaluation of I_j,ss_ (pA) related to a typical cell pair cultured for 72 h in differentiation medium (DM, white bars on the left side of each graph) or to a cell pair cultured for 72 h in DM + PRP (grey bars on the right). For clarity, only the current amplitude values obtained in response to a representative voltage pulse to +130 mV is reported in A, and to −30 mV in B. The current value recorded from the myofibroblast pair (DM 72 h) resulted clearly reduced after the acute addition of heptanol (HEPT, 1 mM) to the bath solution (HEPT DM 72 h). The current flowing through the GJs at the steady-state (HEPT-SENS CURRENT DM 72 h) is obtained by subtracting the current values recorded in the presence of heptanol from those recorded in DM alone. In contrast, the current amplitude recorded from the cell pair cultured in DM + PRP after acute addition of heptanol (HEPT DM + PRP 72 h) showed a slight reduction compared to DM + PRP 72h, having the heptanol-sensitive current a very small value (HEPT-SENS CURRENT DM + PRP 72h). Similar results were systematically observed for any voltage step applied in the bulk of the cell pairs investigated, suggesting a very small amount of functional GJs expressed under PRP treatment. (**C**) Characteristic time course of I_j_ (pA) recorded from a cell pair cultured in DM (DM 72 h) that exceptionally showed voltage independent features. (**D**) The same current traces recorded after acute heptanol addition (HEPT DM 72 h). (**E**) Resulting current traces (HEPT-SENS CURRENT DM 72 h) obtained by subtracting currents in D from currents in C, and representing the small heptanol-sensitive flux through the GJs. Note the different ordinate scale in E.

To test for the current really flowing through GJs, we added heptanol (1 mM), a regularly used GJ channel blocker [59], to the bath solution during the recordings. The I_j_ was evoked from a cell pair cultured in DM, and then records were acquired from the same cell pair. After that, heptanol was acutely added to the bath solution. The recorded currents were significantly reduced compared to those elicited without heptanol. The mathematical subtraction of these two sets of traces gave the heptanol-sensitive current that is the one flowing through the GJs. In contrast, heptanol added during the recordings to cell pairs cultured in DM in the presence of PRP usually caused only a slight reduction of the current amplitude, suggesting a minor number of functional GJs allowing the current flow. A typical experiment related to cell pairs cultured in DM or in DM + PRP for 72 h is shown in Figure 6A. Only the current amplitude obtained by applying two representative voltage pulses (+130 and −30 mV) is shown as an example. Similar results were systematically observed for the bulk of cell pairs investigated. Since the heptanol sensitive current had a very small value in DM + PRP 72 h, we suggest a very small amount of functional GJs in the cells cultured in this experimental condition (Figure 6B–D).

Noteworthy, even in those cell pairs cultured in DM for 72 h that exhibited not-voltage-dependent I_j_, we could still measure a heptanol-sensitive current, suggesting that also the not-voltage dependent GJ functionality was hampered by the GJ blocker.

**Figure 7:**
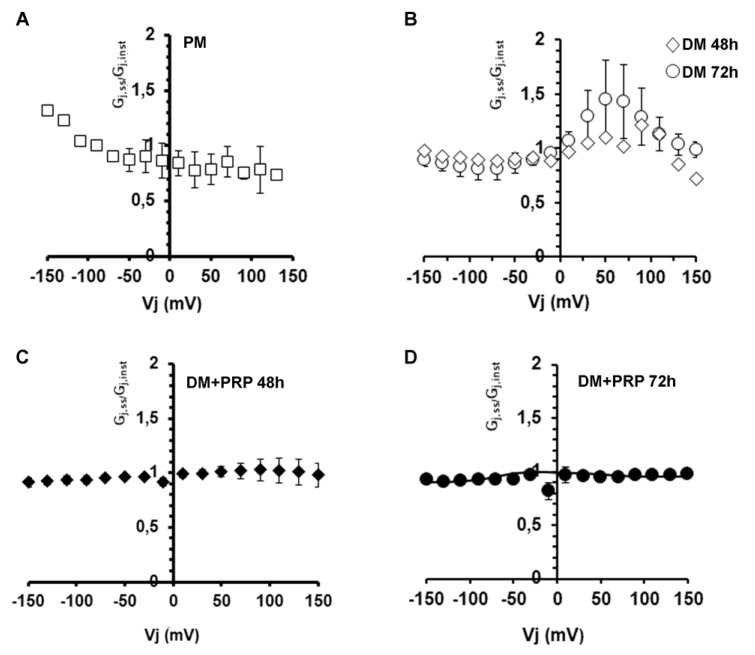
Voltage dependence of the transjunctional conductance G_j_. (**A**,**B**) Voltage dependence of the transjunctional conductance obtained by plotting G_j,ss_/G_j,inst_ versus V_j_ related to (**A**) proliferation medium (PM) condition (open squares, *n* = 6), (**B**) differentiation medium (DM) condition at 48 h (open squares, *n* = 9) and 72 h (open circles, *n* = 9). These data obtained in DM show an asymmetrical distribution that becomes more bell-shaped for positive V_j_. The treatment for 72 h gave higher values compared to 48 h, although not statistically significant (*p* > 0.05, multiple unpaired Student’s t-test). (**C**,**D**) Symmetrical linear distribution observed under the concomitant treatment in DM + PRP at **(C)** 48 h (filled squares, *n* = 9), and (**D**) 72 h (filled circles, *n* = 14). All values represent mean  ±  S.E.M. Error bars are visible if they exceed the symbol size.

Finally, we analyzed the conductive properties of GJs. Intercellular current flow in cell pairs of fibroblasts and myofibroblasts were also used to study the dependency of the gap junctional conductance, G_j_, on V_j_ by means of the G_j_-V_j_ plot analysis. The related results are shown in Figure 7. 

Data points obtained from cell pairs cultured in PM (Figure 7A) showed a horizontal distribution for positive V_j_, suggesting the involvement of not-voltage-dependent GJs, in contrast to the not-linear distribution observed for negative V_j_ values. This asymmetrical voltage dependence of the G_j_ can suggest the prevalence of heterotypic GJs in proliferating fibroblastic cell pairs. These data points obtained from the cell pairs cultured in DM for 48 h (Figure 7B) did not follow a merely symmetrical relationship, being almost linear for negative V_j_ and more bell-shaped for positive V_j_. Again, this may be consistent with the expression of more than one Cx isoform in myofibroblasts (possibly assembling in different combinations compared to those observed in PM) and hence confirms the involvement of heterotypic GJ channels in this cell population. After 72 h in DM, the G_j_-V_j_ plot showed more or less the same shape as 48 h, but the G_j_ values for positive V_j_ resulted higher, suggesting a major contribution of the voltage-dependent component. In contrast, G_j_-V_j_ plots related to the cells cultured in DM + PRP both at 48 h and 72 h were symmetrical and linear in any case, suggesting a lack of voltage-dependent GJs. This result suggests the involvement of homotypic GJ channels in this cell population. 

### 3.3. PRP Reduces Cx43 Expression and Increases Cx26 Expression in Differentiated Myofibroblasts

Since the electrophysiological experiments showed an increase of I_j_ and G_j_ functionality during myo-differentiation of fibroblasts, we cultured the cells in DM in the presence of the GJ channel blocker heptanol (1 mM) for 72 h, to test the effective involvement of GJs in myofibroblast generation. We first analyzed the expression of α-sma in this experimental condition. As assessed by Western blotting and confocal immunofluorescence analyses, the cells exposed to DM + heptanol exhibited a clear reduction of α-sma expression (Figure 1A,B,F,G,I) compared to control differentiated myofibroblasts cultured in DM, supporting a key role of the GJs in the acquisition of myofibroblastic phenotype. Of note, the cells cultured in DM + PRP showed a more robust reduction of α-sma then those cultured in DM + heptanol, likely suggesting that PRP-induced prevention of fibroblast myofibroblast differentiation involves the activation of multiple molecular mechanisms. The cells cultured in DM + heptanol + PRP showed a significantly reduced (*p* < 0.05) expression of α-sma as compared to that observed in the cells exposed to single treatment (i.e., DM + heptanol or DM + PRP). 

Then, taking into consideration the increasingly marked voltage dependence of the I_j_ recorded during differentiation time, we performed experiments aimed to evaluate the expression of the typical Cx types forming voltage- dependent connexins, namely Cx43.

**Figure 8:**
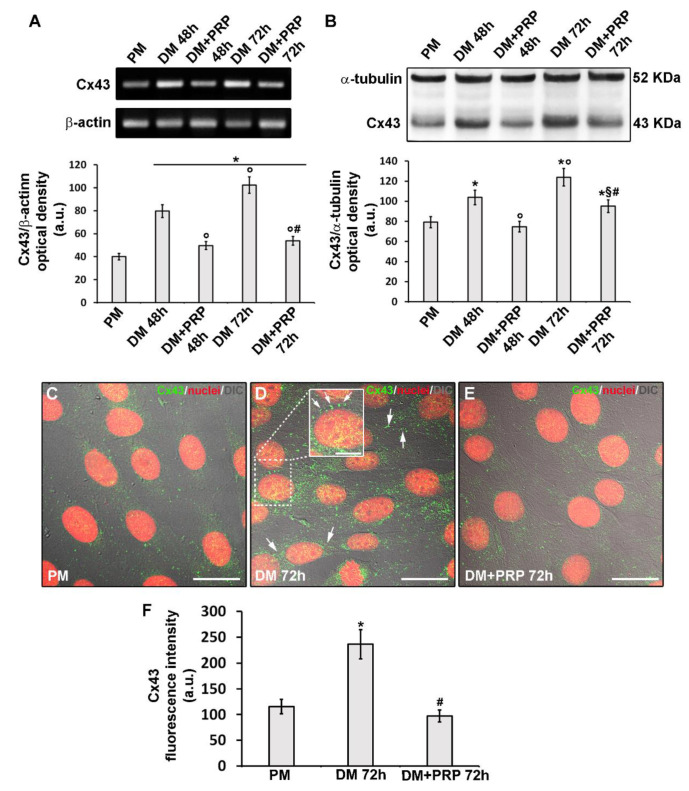
Cx43 expression and localization during fibroblast to myofibroblast transition and related PRP effects. Fibroblasts were induced to differentiate into myofibroblasts by culturing in differentiation medium (DM) in the presence or absence of PRP for 48 h and 72 h. The cells cultured in proliferation medium (PM) were used as control undifferentiated cells. (**A**) RT-PCR analysis of Cx43 expression in the indicated experimental conditions. Representative agarose gel is shown. The densitometric analysis of the bands normalized to β-actin is reported in the histogram. (**B**) Western Blotting analysis of Cx43 expression. Histogram showing the densitometric analysis of the bands normalized to α-tubulin. (**C**–**E**) Representative superimposed differential interference contrast (DIC, grey) and confocal fluorescence images of the cells immunostained with antibodies against Cx43 (green) and counterstained with propidium iodide (PI, red) to label nuclei. Scale bar: 30 µm. Scale bar in the inset in D: 15 µm. Arrows indicate the localization of Cx43 at the membrane level of two adjacent cells. (**F**) Histogram showing the densitometric analysis of the intensity of the Cx43 fluorescence signal performed on digitized images in 20 regions of interest (ROI) of 100 μm^2^ for each confocal stack (12). Data shown are mean ± S.E.M. and represent the results of at least three independent experiments performed in triplicate. Significance of difference: * *p* < 0.05 versus PM; ° *p* < 0.05 versus DM 48 h; # *p* < 0.05 versus DM 72 h; § *p* < 0.05 versus DM + PRP 48 h (One-way ANOVA followed by the Tukey post hoc test).

We found that Cx43 expression both at mRNA and protein levels significantly increased (*p* < 0.05) with time in the cells cultured in DM as compared to control cells in PM as judged by RT-PCR (Figure 8A) and Western blotting analyses (Figure 8B), respectively. 

**Figure 9:**
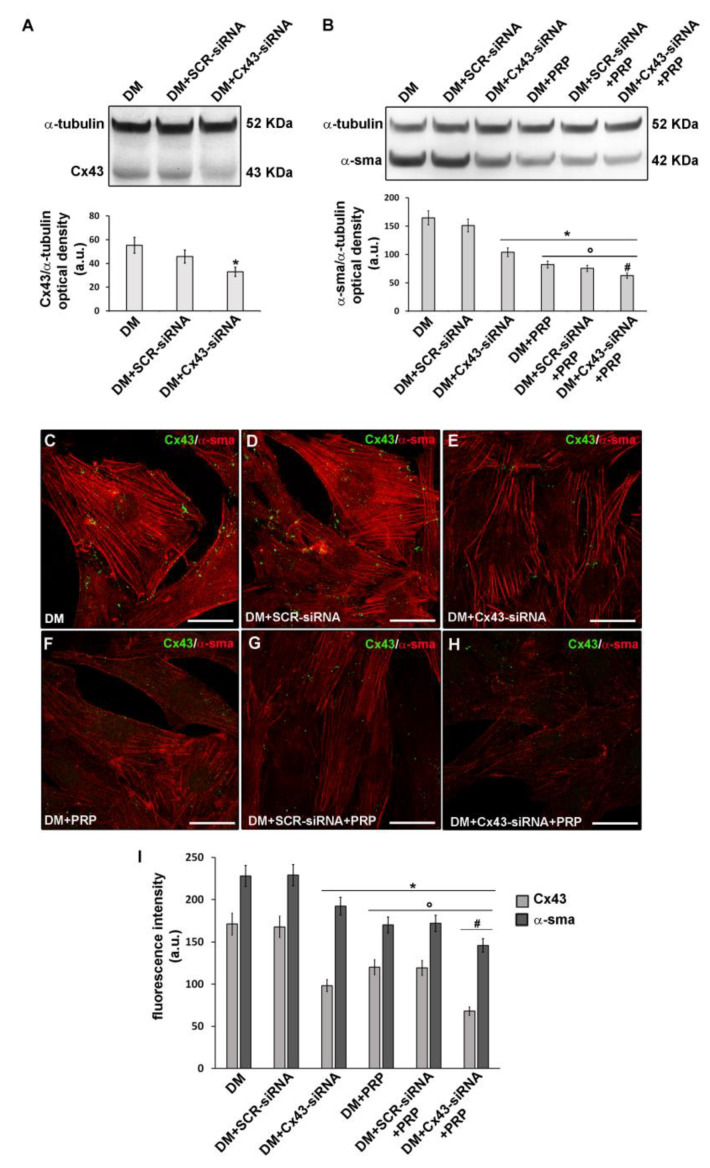
Effect of inhibition of Cx43 expression on fibroblast to myofibroblast transition and related PRP effects. Fibroblasts were silenced by specific Cx43-siRNA duplexes and cultured in differentiation medium (DM) for 48 h in the absence or presence of PRP. SCR-siRNA duplexes were used as an internal control. (**A**,**B**) Western Blotting analysis of (**A**) Cx43 and (**B**) α-sma expression. The densitometric analysis of the bands normalized to α-tubulin is reported in the histograms. (**C**–**H**) Representative confocal fluorescence images of the cells double immunostained with antibodies against Cx43 (green) and α-sma (red). Scale bar: 25 µm. **(I)** Histogram showing the densitometric analysis of the intensity of Cx43 and α-sma fluorescence signal performed on digitized images in 20 regions of interest (ROI) of 100 μm^2^ for each confocal stack (12). Data shown are mean ± S.E.M. and represent the results of at least three independent experiments performed in triplicate. Significance of difference: * *p* < 0.05 versus DM; ° *p* < 0.05 versus DM + CX43 − siRNA; # *p* < 0.05 versus DM + PRP (One-way ANOVA followed by the Tukey post hoc test).

Confocal immunofluorescence analysis confirmed the increase of Cx43 expression in differentiated cells after 48 h (data not shown) and even more after 72 h of culture in DM as compared to control undifferentiated cells in PM (Figure 8C,D,F). Moreover, we demonstrated the protein localization either at the cytoplasmic level or at the cell membrane level of adjacent cells (Figure 8C,D). To confirm the key role of this Cx isoform in fibroblast to myofibroblast transition, we silenced the cells for the expression of Cx43 by specific siRNA before culturing them in DM for 72 h (Figure 9A).

These cells exhibited a significant reduction (*p* < 0.05) of α-sma expression (Figure 9B,C,D,E,I) compared to cell cultured in DM, suggesting that Cx43 was required for the differentiation process. Of note, the cells cultured in DM + PRP concomitantly to reduced α-sma, showed a significant reduction of Cx43 expression (Figure 8A,B,E,F). This outcome was consistent with the electrophysiological data showing the reduction of voltage-dependent responses in these cells. Notably, according to the results of the experiments achieved in cells cultured with heptanol (Figure 1), the cells cultured in DM + PRP showed reduced expression of α-sma with respect to the cells silenced for Cx43 cultured in DM (Figure 9 B–I). Cells silenced for Cx43 expression and exposed to DM + PRP exhibited reduced α-sma expression levels as compared to those observed in the cells exposed to single treatment (i.e., DM + siRNA or DM + PRP) (Figure 9 B–I). 

**Figure 10:**
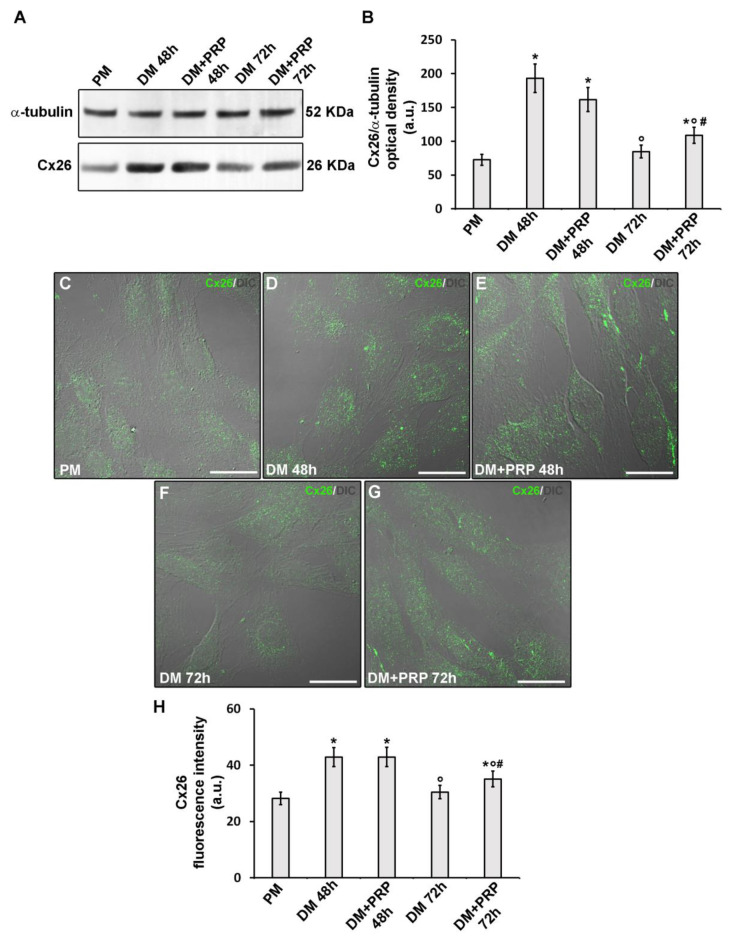
Cx26 expression during fibroblast to myofibroblast transition and related PRP effects. Fibroblasts were induced to differentiate into myofibroblasts by culturing in differentiation medium (DM) in the presence or absence of PRP for 48 h and 72 h. The cells cultured in proliferation medium (PM) served as control undifferentiated cells. (**A**,**B**) Western Blotting analysis of Cx26 expression. (**A**) Representative blot. (**B**) Histogram showing the densitometric analysis of the bands normalized to α-tubulin. (**C**–**G**) Representative superimposed differential interference contrast (DIC, grey) and confocal fluorescence images of the cells immunostained with antibodies against Cx26 (green) showing the cellular localization of the protein. Scale bar: 25 µm. (**H**) Histogram showing the densitometric analysis of the intensity of the Cx26 fluorescence signal performed on digitized images in 20 regions of interest (ROI) of 100 μm^2^ for each confocal stack (12). Data shown are mean ± S.E.M. and represent the results of at least three independent experiments performed in triplicate. Significance of difference: * *p* < 0.05 versus PM; ° *p* < 0.05 versus DM 48 h; # *p* < 0.05 versus DM 72 h (One-way ANOVA followed by the Tukey post hoc test).

Finally, even the occurrence of a not-voltage-dependent response in myofibroblast pairs (although in a minority) as well as of the increase of this type of response after treatment with PRP, we analyzed the expression of a typical Cx type forming not/scarcely voltage-dependent connexons, namely Cx26. Western blotting (Figure 10A,B) and confocal immunofluorescence (Figure 10C–H) analyses demonstrated that Cx26 expression significantly increased in the cells after culture in DM for 48 h with respect to undifferentiated cells cultured in PM (*p* < 0.05). By contrast, the cells cultured in DM for 72 h exhibited Cx26 expression levels comparable to those of undifferentiated cells. The cells cultured in DM for 48 h in the absence or presence of PRP exhibited comparable levels of Cx26 expression (*p* > 0.05). Of note, the cells cultured in DM + PRP for 72 h exhibited a slight but significant increase (*p* > 0.05) of Cx26 as compared to cells cultured in the absence of PRP (Figure 10A,B,F,G,H).

## 4. Discussion

In recent years, great attention has been paid to the identification of new therapeutic agents and treatments that may promote the repair/regeneration of damaged skeletal muscle. In such a context, several in vitro and in vivo studies provided evidence supporting the advantage of the use of PRP for muscle regenerative purpose [30,66]. In this line, we have recently demonstrated the capability of PRP to either stimulate proliferation and differentiation of myogenic progenitors, including satellite cells [58], or prevent the TGF-β1 induced differentiation of fibroblasts towards myofibroblasts [48,57].

These data led us to suggest that PRP, if properly administered along the cascade of events through which skeletal muscle repair/regeneration proceeds (which also includes the physiological fibrotic reparative response), could exert a double beneficial effect on the healing of injured muscle. This may consist in the direct activation of the resident cells effectors of muscle regeneration, responsible for the formation of new muscle fibers and, in parallel, in the modulation/prevention of an excessive fibrotic response, thus contributing to the recreation of a more hospitable and conducive microenvironment for muscle progenitor functionality and thus the promotion of tissue regeneration. Experiments are ongoing in our lab aimed to assess the effects of PRP on differentiated myofibroblasts, by evaluating the capability of this blood product to modulate their fate. The results should be of interest to support the anti-fibrotic action of PRP. However, the ability of PRP in antagonizing fibrotic signaling pathways is still an issue of debate. Some reports show limited effectiveness or even inefficacy of this blood-derived product in counteracting the skeletal muscle fibrotic response [30,42,44,67,68,69,70,71,72,73,74,75]. The great heterogeneity of the available PRP formulation, PRP dosage and application timing represent critical points that may account for the reported conflicting results concerning the effects of this blood product in the modulation of skeletal muscle tissue fibrosis.

Based on these considerations, studies aimed to support the anti-fibrotic effect of this blood product are needed, as well as researches focused on the identification of the cellular and molecular target mechanisms of PRP, underpinning its action. 

### 4.1. PRP Counteracts Myofibroblast Generation

According to findings from our previous studies and other research groups [30,47,48,76,77], here we have confirmed the ability of PRP to counteract the core cellular process of the fibrotic response, namely differentiation of fibroblasts towards myofibroblasts induced by the pro-fibrotic agent TGF-β1, based on: i) morphological and biochemical analyses showing that the cells treated with TGF-β1 in the presence of PRP did not acquire a mature myofibroblastic phenotype; indeed they rather appeared more spindle-shaped and showed either a reduction of type-1 collagen expression and a lower expression of α-sma, that was also less organized in filamentous structure as compared to differentiated cells; ii) the novel electrophysiological recordings of the membrane passive properties and gap junctional functionality, showing the ability of PRP to modify such parameters with respect to those recorded in differentiated myofibroblasts. Particularly, in the present experiments we observed that differentiated myofibroblasts tended to have a more positive RMP and PRP treatment counteracted this occurrence. The RMP is always critical for cell function since any small alteration of its value can substantially change cell excitability, contractility, and other properties, such as cell migration [21]. The less positive membrane potential registered in the cells induced to differentiate in the presence of PRP may counteract the depolarization of myofibroblasts and hamper their contractility, leading to an altered functionality. In this regard, it was shown that depolarization causes enhancement of ventricular myofibroblast contractility [21]. In this view, the present findings may suggest that PRP can revert myofibroblast RMP towards a more ‘dormant’ condition, counteracting their full differentiation. 

In addition, PRP action opposed the increase of R_m_ value observed in differentiating conditions, showing its ability to revert the effect on the resting conductive properties induced by TGF-β1. The R_m_ parameter, corresponding to the reciprocal value of the membrane conductance, G_m_, gives an idea of the total resting ionic fluxes across the membrane; thus, its physiological relevance is strictly linked to cell excitability. Moreover, the C_m_ value assumed as an index of membrane surface area increased in the cells induced to differentiate as compared to proliferating cells. This observation was in agreement with the morphological analysis showing that the cells tended to increase their size upon TGF-β1-induced differentiation. Both phenomena were counteracted by PRP. Notably, the latter data are in accordance with the electrophysiological results described in our previous report dealing with the anti-fibrotic potential of relaxin [23].

### 4.2. Role of GJIC and Cx43 in Myofibroblast Generation

The main relevance of our study is the contribution toward defining the molecular and functional mechanisms regulating TGF-β1 induced fibroblast-myofibroblast transition, highlighting the role of GJs in this process as well as the involvement of voltage-dependent connexin isoform, namely Cx43. In particular, we found that the majority of differentiated myofibroblast pairs exhibited an enhancement of I_j_ amplitude in the course of differentiation, suggesting an increased functionality of GJs, especially of the voltage-dependent ones, with increasing exposure time to TGF-β1. The role of GJs in this differentiation process was confirmed by the use of heptanol, a common GJ blocker. When the cells were induced to differentiate in the presence of heptanol, they actually failed to acquire a myofibroblast phenotype, indicating an essential role of functional GJs in the promotion of fibroblasts differentiation towards myofibroblasts. These data are in good accordance with previous studies showing that the selective blockade of GJs downregulated myofibroblastic phenotype [78,79]. Therefore, it can be stated that GJIC is of crucial importance in our cell model to regulate the fibroblast transformation towards myofibroblast. However, a possible role of such intercellular communications in the functional coupling of mature myofibroblasts to coordinate their activity can also be speculated [18,51,52,53,54]. In fact, while it is well accepted that myofibroblasts are responsible for the reparative scar formation and contraction, it is not clear yet whether they act individually or behave synchronically [78]. In this regard, we can propose that myofibroblasts can, at least in part, act as a coordinated functional syncytium, thus that hindering intercellular communication may represent a therapeutic target in diseases characterized by an overabundance of these contractile cells. Another important point is the ability of myofibroblasts to interact, by GJs, with other resident cell types of tissue globally affecting the organ functionality [80,81,82]. The analysis of the transjunctional conductance, G_j_, gave some interesting information. Usually, the higher the G_j_, the faster the current flows from a cell to the adjacent one, resulting in faster propagation speed [83]. The estimated G_j_ is the overall result of the total number of GJ channels docked between cells, the single-channel conductance of each GJ channel, and their functional states (fully open, sub conductance, or closed states). The GJ functional states can be dynamically modulated by chemicals and transjunctional voltage. The transjunctional voltage-dependent gating is an intrinsic property in all characterized GJs. In this study, we found that the G_j_ in differentiated myofibroblast pairs showed a progressively more marked voltage-dependence, suggesting a prevalent expression of voltage-dependent Cxs in myofibroblasts. From a functional point of view, this may reflect the need for myofibroblasts to be coupled in response to stimuli that cause membrane potential alterations. However, myofibroblasts likely need to express a kind of Cx, such as Cx43, whose trafficking, half-life, and regulation (by phosphorylation) can be maximally modulated during differentiation to myofibroblast [84]. Corroborating this suggestion coming from electrophysiological records, here we found that myofibroblasts showed an increased expression of Cx43. Furthermore, we found that cells silenced for the expression of Cx43 did not exhibit a mature myofibroblastic phenotype when cultured in differentiation condition in the presence of TGF-β1, suggesting the requirement of Cx43 for fibroblast-myofibroblast transition. Collectively these data are in accordance with the findings of our recent study [48] demonstrating an upregulation of mRNA expression of Cx43 in TGF-β1 treated cells (i.e., myofibroblasts). As well they agree with the studies by Asazuma-Nakamura and co-workers (2009) [85] and by Paw and co-workers (2017) [86] showing that Cx43 positively regulated myofibroblastic differentiation of cardiac and bronchial fibroblasts, respectively. In parallel, other studies showed that Cx43 expression is largely modulated during wound repair, and the modulation of Cx43 expression and gap junctional communication can be beneficial to wound healing [87]. This process can be definitely altered by modulating Cx43 expression: wound closure can be delayed when Cx43 is overexpressed or accelerated when the levels of epidermal Cx43 are reduced [88,89,90,91]. In line with this, the transient blockade of Cx43 functions has been shown to reduce fibrosis as well as to promote experimental wound healing [89], and the normal GJ functions of Cx43 seems to be important for normal fibroblast function [92]. In this regard, multiple clinical trials are investigating Cx43 modulators and specific peptides targeting the intracellular loop, and the C-terminal tail region of this protein appear promising [93]. Based on our present findings, showing an increase of Cx43 expression in differentiated cells not only at the plasma membrane level but also in the cytoplasm, it is worth mentioning that a GJ independent function of Cx43 during fibroblast to myofibroblast transition cannot be excluded [59,94]. Taking into account the reported channel-independent influence of Cx43 on cytoskeleton remodeling and cell migration, we may speculate a similar role [94] for stress fiber assembly during fibroblast- myofibroblast transition. 

### 4.3. GJs, Cx43, and Cx26 as Molecular Candidate Targets of the Potential Anti-fibrotic Action of PRP

Another main finding of this study is the compelling experimental evidence indicating, for the first time, that GJs and Cx43 are molecular candidate targets of the potential anti-fibrotic action of PRP. Indeed, PRP treatment affected the occurrence of voltage-dependent I_j_, which was reduced at 48 h and even abolished after 72 h of culture; concomitantly, it prevented the TGF-β1 induced increase of Cx43 expression. Interestingly, the cells induced to differentiate in the presence of PRP mostly exhibited scarcely/not voltage-dependent I_j_ along with an observed upregulation of Cx26, especially after 48 h. We may suppose that these events reflect a compensatory mechanism to maintain a sort of cell-to-cell communication, upon PRP treatment. In addition, we may speculate a major role of Cx26 in this condition that may be linked to the ability of this Cx type to form hemichannels rather than highly regulated GJs. We should point out the ability of hemichannels themselves to act as membrane channels able to mediate cell communication with surrounding extracellular environment: recent studies confirm a link between hemichannel-mediated ATP release and the progression and development of fibrosis in different tissue types [95,96,97,98]. In this line, the role of Cx26 in forming scarcely voltage-dependent GJs and/or hemichannels and the possible relation with ATP content in our cell model is an interesting topic deserving further investigation.

### 4.4. Factors Released by PRP Possibly Modulating Cx Expression and GJ Functionality During Myofibroblast Generation

Finally, it is worth mentioning that the factors released by PRP possibly modulating Cx expression and GJ functionality during myo-differentiation process remain to be identified as well as their potential modes of action. We have previously demonstrated that PRP contains vascular endothelial growth factor (VEGF)-A and that, through this factor linking its receptor-1 (VEGFR-1 or Flt-1), PRP antagonizes TGF-β1/Smad3, thus preventing fibroblast myo-differentiation [30,48]. Taking into consideration that VEGF may modulate the expression of Cx43 and/or GJ functionality in different cell types [99,100,101] we may speculate that GJs/Cx43 might represent a downstream target of VEGF-A/VEGFR-1 mediated signaling in our experimental cell model. Studies are ongoing in our lab to assess this hypothesis. Moreover, it is known that GJ assembly and disassembly are events highly regulated by a sequence of protein kinase activation and phosphorylation events. This kind of regulation, as extensively reported for Cx43, has a net effect of reducing GJ communication [102,103,104]. Therefore, we may also postulate that VEGF-A or other factors released by PRP may reduce GJ/Cx43 functionality affecting such phosphorylation events. In such a view it has been reported that insulin-like growth factor (IGF)-1 is able to decrease gap junctional communication by inducing activation of PKCγ, enhancing the interaction between PKCγ and Cx43 and the phosphorylation of Cx43 by PKCγ [105] in epithelial cells. Since IGF-1 has been reported to be contained in PRP [106] and has been supposed as a potential modulator of fibrogenic events and pathways [14,107,108], it is tempting to speculate that similar interactions may also occur in our cell system. Therefore, the capability of PRP to counteract fibroblast differentiation towards myofibroblasts and its ability to modulate GJIC could also involve the IGF-1 signaling pathway. A full characterization of the releasing profile of likely cross-talking factors present in PRP are required to understand the molecular mechanisms underpinning the action of this plasma product. 

## 5. Conclusions

In conclusion, the results of the present in vitro study provide the first experimental evidence that upregulation of Cx43 and the parallel increase of voltage-dependent GJ functionality are important mechanisms by which TGF-β1 endorses fibroblast differentiation towards myofibroblast, and that PRP treatment hampers this effect. 

The main limitations of this study rely on the in vitro experimentation on the NIH/3T3 cell line. Obviously, the in vitro experimentation eliminates many paracrine/juxtacrine mechanisms, possibly regulating in situ intercellular interactions and cell functionality as well as the mechanical forces exerted by the surrounding microenvironment, including ECM stiffness, affecting cell behavior [16,18,109]. NIH/3T3 cells represent a widely used, reliable model to study fibroblast biology. We previously demonstrated that these cells show similar behavior, in terms of differentiation marker expression and electrophysiological parameters, to primary fibroblasts, such as human dermal fibroblasts, skeletal, and cardiac fibroblasts [23,48,55,56]. Nevertheless, the growing evidence on functional heterogeneity and the origin-linked response of fibroblasts to stimuli must be taken into account [110,111,112]. Therefore, we acknowledge that a different experimental set using primary cultures of skeletal muscle-derived fibroblasts could have offered in vitro findings possibly more closely related to in vivo conditions of skeletal muscle disease and fibrosis. 

Another limitation is represented by the lack of a full characterization of the growth factors released by PRP. This should be relevant to understand better PRP mechanisms of action in the modulation of fibroblast-myofibroblast transition and to achieve a therapeutic translation of this approach. Furthermore, standardization of PRP preparation techniques as well as application protocols would allow performing meaningful comparative analyses.

However, despite these aspects, this research contributes to add further insights into molecular and functional mechanisms regulating fibroblast-myofibroblast transition. It likewise supports the anti-fibrotic action of PRP by means of its ability to hamper myofibroblast generation, targeting GJs, thus providing cues to novel therapeutic targets. 

## Figures and Tables

**Table 1:** Electrophysiological analysis of the membrane passive properties.

	PM	DM 48 h	DM + PRP 48 h	DM 72 h	DM + PRP 72 h
RMP (mV)	−62.0 ± 6.8 (*n* = 6)	−41.7 ± 3.75 (*n* = 5)	−50.2 ± 8.9 (*n* = 7)	−45.16 ± 4.06 (*n* = 7)	−47.37 ± 3.1 (*n* = 6)
R_m_ (MῺ)	159.2 ± 31.0 (n = 6)	353.8 ± 85.9 (n = 5)	164.7 ± 17.3 (n = 14)	585.3 ± 100.9 *^,#^ (n=11)	436.2 ± 71.3 *^,#^ (n=15)
C_m_ (pF)	7.05 ± 1.2 (*n* = 6)	23.5 ± 4.7 * (*n* = 9)	6.82 ± 0.5 ^§^ (*n* = 9)	17.6 ± 2.9 *^,#^ (*n* = 8)	8.3 ± 0.9 (*n* = 14)

Data are reported as mean ± S.E.M. * *p* < 0.05 versus PM; ^#^ *p* < 0.05 versus DM + PRP 48 h; ^§^ *p* < 0.05 versus DM 48 h (one-way ANOVA, followed by Bonferroni’s post hoc test). The number of investigated cells is indicated by “*n*” in brackets for each condition.
